# Multifaceted sleep health score is associated with frailty in a national sample of older adults in Taiwan: Sex matters

**DOI:** 10.1093/geroni/igab046.3714

**Published:** 2021-12-17

**Authors:** Tuo Yu Chen, Soomi Lee, Orfeu Buxton

**Affiliations:** 1 Taipei Medical University, Taipei, Taipei, Taiwan (Republic of China); 2 University of South Florida, Tampa, Florida, United States; 3 The Pennsylvania State University, The Pennsylvania State University, Pennsylvania, United States

## Abstract

Although individual sleep characteristics are related to frailty, these characteristics do not occur separately. A multidimensional measure of sleep might provide a better estimation of frailty compared to isolated sleep characteristics. This study investigated the association of a multidimensional measure of sleep health with frailty and examined whether such relationship differed by sex. Data were from the Taiwan Longitudinal Study on Aging (2011), a survey with a nationally representative sample of Taiwanese older adults (N=2,015). Frailty was defined using the Fried-criteria. Self-reported sleep during the past month was used to conceptualize the five sleep health dimensions in the SATED model (satisfaction-alertness-timing-efficiency-duration; higher scores representing better sleep health). Their relationship was estimated using logistic regression analysis adjusting for sociodemographic (age, sex, education), health (chronic conditions, cognitive function, pain, depressive symptoms [excluding items overlapping with frailty and sleep]), and lifestyle (drinking, smoking, exercise) characteristics. The results showed that having a better sleep health composite score was significantly related to lower odds of being frail in both sexes adjusting for sociodemographic information. Such effect remained significant among females but not males after adding health and lifestyle characteristics to the models. Sleep satisfaction and daytime alertness in both sexes and sleep duration among females were significantly associated with frailty adjusting for sociodemographic information. Only alertness among males was significantly related to frailty in model with all covariates. Our findings show that having a better sleep health across multiple dimensions is related to a lower risk of being frail, and the association differs by sex.

